# Anthropologie de la Mort Infectieuse

**DOI:** 10.48327/mtsibulletin.2021.107

**Published:** 2021-06-05

**Authors:** P. Charlier

**Affiliations:** Laboratoire anthropologie archéologie biologie (LAAB), Université Paris-Saclay & Musée du quai Branly - Jacques Chirac, 2 avenue de la source de la Bièvre, 78180 Montigny-Le-Bretonneux, France

**Keywords:** Rituels funéraires, Anthropologie funéraire, Ethnologie, Épidémiologie, Paléopathologie, Funeral rituals, funerary anthropology, ethnology, epidemiology, paleopathology

## Abstract

Comment s'adaptent les rituels funéraires lorsque le décès est d'origine infectieuse, ou lorsque la mort survient en contexte épidémique? Quelles modifications sont nécessaires? Quels fondamentaux anthropologiques sont écornés, détournés ou pris à contre sens? On verra, dans les exemples pratiques de terrain et historiques choisis, comment la communauté des vivants s'arrange avec ses peurs et ses exigences métaphysiques vis-à-vis de la communauté des défunts, composant avec les codes et les croyances. Bref, comment s'établissent des rituels parallèles permettant de contenter les deux parties.

## Abstract (en)

How do funeral rituals adapt when death is of infectious origin, or when death occurs in an epidemic context? What changes are needed? What anthropological fundamentals are altered, diverted or reversed? We will see, in the following anthropological and historical examples, how the community of the living deals with its fears and metaphysical demands vis-à-vis the community of the deceased, dealing with codes and beliefs. In short, how parallel rituals have been established to satisfy both parties.

## Contexte Anthropologique

Tout rituel a été mis en place pour « organiser le chaos », pour borner la prise de décision des membres d'une communauté lorsque survient un évènement potentiellement déstabilisant, soit positif (naissance, mariage, récolte, semailles, etc.), soit négatif (accident, maladie, famine, décès, etc.) [6].

Qu'en est-il lorsque la mort survient en contexte épidémique? Lorsqu'une infection touche de façon diffuse une population, et que celle-ci est obligée de s'en prémunir par des procédures extrêmes touchant à la fois les vivants et les morts?

Il convient d'abord de comprendre et mettre en place une anthropologie des rites de mort. Dans un livre issu d'un entretien accordé au géographe Michel Lussault, l'anthropologue Maurice Godelier expose la succession de trois moments et trois réponses sociales à ces périodes critiques [10].

Le premier: au moment de l'agonie, quelle attitude avoir vis-à-vis de la personne qui décède? Soit on s'abstient de toute manifestation de tristesse (en Chine du Sud, par exemple), soit on manifeste bruyamment sa peine (chez les Baruya de Papouasie-Nouvelle-Guinée, par exemple).

Le deuxième: lorsque l'individu a « fini de mourir », il reste encore à disposer de son corps, c'est-à-dire à gérer le cadavre, soit en l'inhumant, soit en l'exposant, soit en l'incinérant, soit même en le consommant. Puis, vient enfin le troisième et dernier moment: dans toute société, il y a l'obligation, pour les vivants, de faire, sous des formes diverses et pour des temps plus ou moins longs, le deuil du défunt. Ces trois invariants, les deux premiers, spéculatifs, et le troisième, opératif, constituent une grille d'analyse utile pour aborder les formes diverses de rapport(s) à la mort dans les sociétés.

Dans un autre ouvrage, le même Maurice Godelier pose l'existence de deux invariants concernant la mort *stricto sensu*, que l'auteur a mis au jour en comparant 14 religions du passé ou du présent, tribales ou non, monothéistes ou polythéistes [9].

Le premier est de portée universelle: la mort ne s'oppose pas à la vie, mais à la naissance. Ainsi, la mort n'est pas l'anti-vie, mais la sortie de la vie, comme la naissance est l'entrée dans la vie. Ce sont deux extrémités d'un long moment, mais pas l'opposé de la vie.

**Figure 1:**
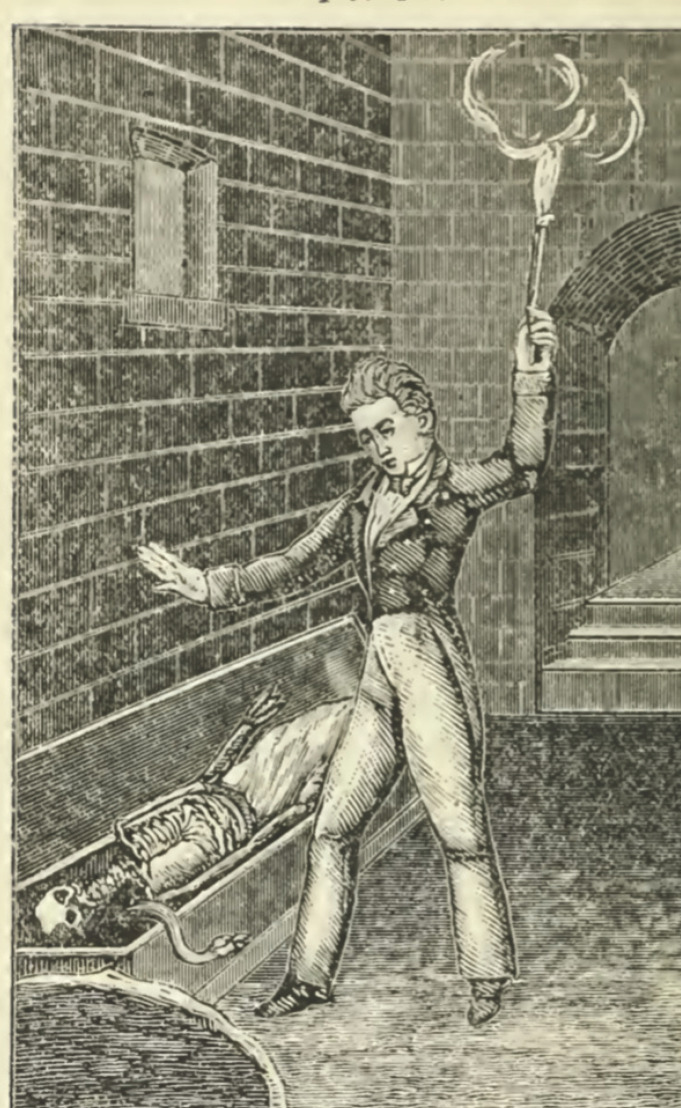
Planche XI du Miroir des âmes ou exposition des différents états des âmes par rapport à Dieu (1829). Le squelette gisant dans le cercueil est celui d'une ancienne amante morte de syphilis Plate XI of the Miroir of Souls or Exposition of the Different States of Souls in Relation to God (1829). The skeleton lying in the coffin is that of a former lover who died of syphilis

Le second est de portée plus restreinte: il existe, pour beaucoup de sociétés, l'idée d'un jugement *post-mortem*, c'est-à-dire l'idée que nos actions passées détermineront peu ou prou le devenir de notre âme ou de ses avatars dans un au-delà. Ce que sous-tend ce concept, c'est la nécessité, d'abord, de mener une vie « bonne » (comme un investissement sur un avenir lointain, plus ou moins certain selon sa croyance religieuse), mais aussi le besoin de se préparer à sa propre mort (c'est-à-dire au jugement dernier, par l'entremise d'un *Livre des morts* ou du *Texte des Pyramides*, si l'on appartient à l'élite pharaonique, ou des traités du bien mourir, ces *Ars moriendi* de l'Occident médiéval et jusqu'au 19^e^ siècle) [4] (Fig. 1).

Ces invariants sont supposés indifférents au fait que la mort soit celle d'un parent ou d'un ennemi, d'un malade ou d'une victime d'assassinat, etc. Mais qu'en est-il réellement? En l'absence de suffisamment d'argent, un Égyptien antique ne pouvait bénéficier d'un papyrus psychopompe, d'amulettes protectrices contre les démons voleurs d'âme, ou même d'un embaumement permettant de vivre l'éternité de l'au-delà dans un corps conservé « pour des millions d'années ». Dans l'Égypte antique, le paradis n'appartient qu'aux nantis; peu de risque d'y croiser un paysan… qu'on remplace par des serviteurs de faïence, les *oushebtis*.

## La Mort en Contexte Infectieux

Dans le contexte des maladies infectieuses, qu'en est-il des rituels de mort en période épidémique? Qu'en est-il lorsque le défunt est touché par une maladie stigmatisante (comme la lèpre) ou hautement contagieuse (comme la peste)? Même si, face à ces invariants anthropologiques, on serait tenté de croire qu'il n'existe aucune possibilité intellectuelle d'y ajouter des données épidémiologiques ou médicales, la réalité est peut-être légèrement différente.

Il est en effet des circonstances où le deuil n'est pas marqué, ou de façon très décalée de la mort biologique. Qu'on pense aux lépreux qui, au Moyen-Age occidental, faisaient l'objet d'une messe de funérailles lorsque la maladie était diagnostiquée, avant leur exclusion du monde des vivants (et leur entrée dans la mort sociale qu'est la stigmatisation et la « vie de paria »…). De même avec les *sadhus*, en Inde, qui, au moment de leur renoncement au monde, assistent impassibles à leurs propres funérailles symboliques, pour montrer leur détachement à la fois métaphysique, mais aussi physique. De même encore, avec les condamnés à mort en France, jusqu'au début du 20^e^ siècle, dont le corps, après l'exécution, était escamoté, retiré à la famille, et inhumé de façon anonyme et sans rituel religieux (ni même laïc) dans un carré réservé du cimetière.

En contexte épidémique, la pérennité des rituels religieux, et même l'accompagnement du malade à l'agonie peuvent être des moments de dangerosité pour les proches et la famille, car ils correspondent à des périodes de contagiosité, et donc de diffusion de la maladie: on en a vu l'importance en Afrique sub-saharienne lors des récentes épidémies d'Ebola. L'anthropologue, au contact des équipes de médecins infectiologues et épidémiologistes, étaient là pour adapter les coutumes, quitte à écorner certains de ces invariants, ou à les retarder, pour le bien commun [12].

Le contexte récent de la pandémie de Covid-19 a rendu ces « rituels impossibles » bien plus réels et compréhensibles pour le grand public, avec cette mise à l'écart systématique de la famille au moment de l'hospitalisation, de l'agonie, du décès et des funérailles [5] Dès lors, comment débuter son travail de deuil quand on n'a pas pris conscience de la disparition du défunt en visualisant directement le cadavre, en le touchant, et lui rendant les derniers hommages? Comment s'assurer du départ définitif du mort vers l'au-delà (et donc pour assurer la tranquillité de la communauté des vivants) quand des rituels funéraires n'ont pas été pratiqués pour des raisons sanitaires (on pense à la toilette mortuaire, aux embaumements, etc. par exemple). Comment se recueillir, à distance du drame, sur la sépulture d'un proche quand son corps a été « noyé » au milieu de tant d'autres dans une fosse commune?

## Archéologie et Anthropologie de la Mort

Pourtant, l'anthropologie a montré que nous sommes tous sauf égaux devant la mort: tous les défunts n'ont pas le privilège de funérailles ou de sépultures: fausse-couches, nouveau-nés, enfants en bas-âge… déposés à la hâte et sans offrande en marge du cimetière, parfois-même jetés dans un puits, une citerne ou des égouts (comme ces 150 squelettes de nouveau-nés retrouvés dans les évacuations des eaux usées du lupanar paléo-chrétien d'Ashqelon, en Israël). Dans ce contexte du Proche-Orient des 4^e^-6^e^ siècles ap. J.-C., le deuil semble ne pas exister véritablement en cas de décès d'un enfant au moment de la naissance (que cette disparition soit naturelle ou… artificielle) [8]: pas de souillure du clan, de la maison, ni des objets du quotidien. Rien. Comme s'il ne s'était rien passé. Le corps de l'enfant est escamoté, il disparaît sans bruit, comme un cauchemar dont on se réveille. Les immatures sont les laissés pour compte des rituels funéraires dans l'Antiquité, car, pour reprendre l'expression de Nicole Belayche, « leur mort n'intéresse pas la cité » [11] Ce cas est-il propre aux périodes anciennes et aux civilisations « du lointain », pour reprendre l'expression de Félix Fénéon? Rien n'est moins sûr… En 2014, ce sont près de 800 squelettes de bébés qui ont été découverts dans une cuve en béton, sans cercueil ni pierre tombale, déposés secrètement par les soeurs du couvent catholique de Bon-Secours, à Tuam, en Irlande; privés de tout rituel de funérailles, ces bébés étaient issus de jeunes mères célibataires tombées enceintes hors mariage et hébergées dans le couvent entre 1925 et 1961…

Peut-on aller jusqu'à dire qu'il y a un moyen métaphysique (ou anthropologique?) de contourner cette suspension du rituel? Une possibilité serait de considérer que l'absence de rituel (funéraire) est lui-même un rituel (spécial, exceptionnel). Prenons appui sur des exemples d'anti-rituels portant sur les corps morts: la privation de sépulture et l'atteinte porté au cadavre dans le but, non d'en absorber l'énergie vitale ou le symbolique de la lignée, mais au contraire d'en salir la mémoire et de le priver des bénéfices spirituels du moment du deuil et au-delà.

Premier exemple, celui de la *damnatio memoriae*, dans l'Antiquité romaine, qui consiste en une décision collégiale du Sénat d'oublier collectivement un défunt honni jugé traitre à la patrie. Ses statues sont martelées au niveau du visage et de l'inscription portant sa titulature et son nom: ainsi, plus aucune identification n'est possible. Le retour à l'anonymat est éternel. Le fait de prononcer son nom est puni d'amende et de châtiments corporels. La maison est rasée ou incendiée, sinon rebâtie. Le cadavre est exhumé ou d'emblée privé de sépulture, et jeté dans le Tibre. Aucun rituel n'est fait dans les formes, ce qui implique, pour le défunt, un empêchement de séjour dans l'au-delà (Champs Elysées pour les héros, Enfers pour le commun des mortels): l'âme, impure, lourde, chargée des méfaits commis pendant la vie humaine, va errer tantôt au contact des vivants, tantôt dans le Tartare, ce séjour des malheureux comparable aux enfers chrétiens. Dans ce cas, la société décide d'une « ultra-mort », elle crée de toute pièce un spectre, un fantôme, un revenant que l'on prive à jamais de repos éternel. Presque aucun des invariants décrit par Maurice Godelier n'est présent, ou alors, ce sont des anti-invariants… ce qui revient peut-être au même?

Second exemple, l'exhumation judiciaire, comme celle du pape Formose, en 897 à l'instigation de son successeur Etienne VI; la momie, sortie du sarcophage, est revêtue de ses atours pontificaux puis portée devant un collège d'évêques romains, assise sur un trône et subit ce qu'on a appelé plus tard un « concile cadavérique »; pour cette parodie de procès, l'avocat du cadavre est un diacre qui doit répondre aux accusations de parjure. L'historien Daniel Rops a laissé une idée assez savoureuse de ce que fut ce procès inéquitable et macabre: « *Une cérémonie abominable suivit, où le mort fut dégradé, dépouillé des vêtements pontificaux auxquels collaient les chairs putréfiées, jusqu'au cilice que portait ce rude ascète; les doigts de sa dextre [main droite] furent coupés, ces doigts indignes [selon ses juges], qui avaient béni le peuple* »… [13] Finalement le cadavre est privé de sépulture, comme au temps de la *damnatio memoriae*, et jeté, comme à l'accoutumée, dans le Tibre …qui pourrait presque finir par ressembler au Gange ! Pour la petite histoire, Etienne VI sera déposé peu après par une révolte menée par les partisans de Formose, le Tibre rendra miraculeusement le corps du défunt pape, et les statues des saints s'inclineront même sur le passage du cortège funéraire pour saluer comme il se doit l'un des leurs.

Formose s'oppose trait pour trait à la légende d'Inès de Castro, exhumée en 1357, soit deux ans après ses funérailles, pour être couronnée reine *post-mortem* par Pierre I^er^ de Portugal: la momie revêtue des regalia, assise sur un trône, aurait alors été honorée par tous les grands du royaume par un baiser sur la main… [1] Rien de vrai dans cette anecdote forgée au 16^e^ siècle, sauf l'opposition symbolique entre la privation d'honneur et la dégradation faites à un cadavre, et la restitution d'un honneur perdu et la réhabilitation faites à un autre cadavre.

## Perspectives

Sans aller forcément jusqu'au cadavre, porter un regard anthropologique sur les épidémies, c'est autant l'occasion de confronter les pratiques autour de l'ethnologie et des maladies infectieuses. Dans le vaudou béninois et haïtien, où aucune mort n'est considérée comme naturelle, dans le territoire bamileke du Cameroun où des autopsies traditionnelles sont pratiquées pour rechercher la cause d'une mort qui ne peut être que criminelle (identifier la trace viscérale d'une action de sorcellerie ou d'un traumatisme surnaturel qui n'aurait laissé aucune trace en surface… avec une prédilection hépatique), l'infection n'arrive pas toute seule, elle est toujours envoyée par un tiers (ancêtre irrité, sorcier, génie veillant au respect des interdits rituels, etc.). C'est un empoisonnement par les microbes, ce sont des miasmes mortifères qui ont été transmis, c'est un poison qui a été distillé subrepticement (un « coup de poudre »), c'est une force vitale (*nyama, ti bon'anj*, etc.) qui a été captée pour affaiblir l'organisme, c'est un équilibre qui a été rompu entre le corps et l'environnement…

Ailleurs, comment gérer d'autres types d'épidémies qui « flirtent » avec un contexte économique et politique instable: par exemple cette vague de « raccourcisseurs ou coupeurs de sexe » en Afrique sub-saharienne [2], ces zombies dont chaque catastrophe en Haïti semble multiplier la prévalence [3], ces infections supposées disparues ou cantonnées à des laboratoires spécialisés et que le dégel du permafrost sibérien libère jusque sur des territoires éloignés (*Pandoravirus* identifié par l'équipe de Jean-Michel Claverie à Marseille) [7].

Quelle place, alors, pour le médecin et pour l'anthropologue? Comment peuvent-ils agir à chacun des stades de développement d'une maladie, fut-elle individuelle ou collective? Au-delà de la vie, quel positionnement adopter vis-à-vis du cadavre, de rituels nécessaires à la cohésion sociétale (y compris dans le cadre d'une défiance quant au défunt ou d'une nécessité de lui témoigner reconnaissance et affection)? Comment faire coïncider des obligations qui parfois s'opposent?

Autant de questions pour lesquelles une seule spécialité, un seul regard ne suffit pas. Des chercheurs d'origines et de spécialités particulièrement diverses sont nécessaires, y compris pour les périodes anciennes qui, loin de constituer une « histoire de la médecine » font « dialoguer les cultures » et dégagent des fondamentaux permettant de mieux comprendre des phénomènes actuels.

## Conflits D'intérêts

L'auteur ne déclare aucun conflit d'intérêt.
